# Energy and macronutrient intake and dietary pattern among school children in Bahrain: a cross-sectional study

**DOI:** 10.1186/1475-2891-10-62

**Published:** 2011-06-05

**Authors:** Nadia Gharib, Parveen Rasheed

**Affiliations:** 1 Nutrition Section/Ministry of Health, Manama, Bahrain; 2 Department of Family and Community Medicine, College of Medicine, Dammam University, Dammam, Saudi Arabia

**Keywords:** Energy and macronutrient intake, food frequency, energy % fat, saturated fat, overweight and obesity, Bahraini school children

## Abstract

**Background:**

Obesity is increasing in Bahrain and there is lack of information on the energy and macronutrient intake of children. The objective of this research was to study the energy and macronutrient intake as well as food frequency pattern of Bahraini school children.

**Methods:**

This is a cross-sectional descriptive study conducted on Bahraini school boys and girls aged 6-18 years from all the 11 populated regions of the country. Data on food intake consisted of a 24-hour dietary recall and was obtained by interviewing a sub-sample of the study population. Information was also obtained through a self-administered questionnaire for the entire sample on the weekly frequency of food items that were grouped into 7 categories based on similarity of nutrient profiles. Dietary analysis was performed using the Nutritionist 5 (First Data Bank Version 1.6 1998).

**Results:**

While the average energy intake of students was close to the Estimated Average Requirements of the UK Reference standards, protein intake substantially exceeded the Reference Nutrient Intake values as did daily sugar consumption. Dietary fiber fell short of the Dietary Recommended Values (UK) and 36%-50% students exceeded the Energy % limits for total fat, saturated fat and cholesterol. The Polyunsaturated: Saturated fat ratio remained at an unacceptable level of 0.6 for girls and boys. While sweets, snacks and regular soda drinks were popular, milk, fruits and vegetables were not commonly consumed.

**Conclusions:**

High sugar consumption, low intake of dietary fiber and high energy % of saturated fat and dietary cholesterol by many Bahraini children, is likely to increase their risk of obesity and cardiovascular diseases in later life. Nutrition education programs in schools should emphasize the importance of healthy balanced diets for growth and health maintenance of children as well as dietary prevention of diseases.

## Background

Increasing evidence suggests that diet and lifestyle in childhood and adolescence have a potential lifelong effect for risks of many chronic diseases such as obesity, coronary heart disease, hypertension, diabetes and certain types of cancer [1-6]. The types of diet linked with chronic diseases are found to prevail before pubertal maturation, and children's dietary patterns change only marginally during pubertal development [7]. Childhood and adolescence are also considered two critical periods in the development of obesity, and hence, the prevention of obesity during this phase of life has been suggested as a public health priority [8,9].

With the progress of socio-economic status in Bahrain in recent years, there has been a substantial shift from traditional healthy foods, to diets consisting of high intake of fat, sugar and red meat [10,11] including western fast food. Research has shown that such diets have contributed to increased energy intake and consequently to increasing obesity in children and adults in this region [12-14]. The dietary habits of school children and adolescents are characterized by low intake of fresh fruits, vegetables and milk and a high intake of carbonated beverages and empty calorie foods [15]. Musaiger and Gregory reported that Bahraini teenagers are a group most susceptible to unsound food habits which places them at risk of an adverse nutritional status [16].

While studies have been conducted on the nutritional status and dietary habits of Bahraini children [12,15-20], there is lack of information on energy and macro- nutrient intake. Such information could guide us to suggest need based changes in the eating habits of children for delaying or preventing the development of chronic diseases. The current study addresses the intake of energy and macronutrients as well as food frequency in Bahraini school children; a subsequent publication will focus on intake of micronutrients.

## Methodology

### Subjects and Methods

This is a cross-sectional descriptive study involving qualitative and quantitative variables. The research was conducted from January/1999 to May/2001. The target population was Bahraini boys and girls of primary, intermediate, and secondary levels studying in public schools of the 11 populated regions of Bahrain.

### Sampling

The sample size was determined as per standard method recommended by the WHO Expert Committee (1995) for studies on nutritional status [21]. By applying the standard formula, the estimated sample size obtained was 2443. The sample size finally chosen was 2594; however 32 students declined to participate in the study giving a response rate of 98.7%. A multi-stage sampling design was chosen that combined multi-cluster and simple random sampling methods. Cluster sampling was used in two successive stages: first, for selection of the number of Bahraini students in proportion to the total number of students enrolled in each region; second for allocation of the schools in each region in proportion to the population size of the region. The allocated number of students from each level was chosen by randomly selecting few students from each class. There were 175 government schools, 88 for girls and 87 for boys enrolling a total of 104,189 Bahraini students (52,885 girls and 51,304 boys) from ages 6 to 18 years. Out of 54,782 primary level, 25,779 intermediate level and 23,628 secondary level students, 1386 (2.5%), 596 (2.3%) and 612 (2.6%) students respectively, were chosen for the study sample. The distribution of primary schools in the different regions ranged from 1 to 11, intermediate and secondary schools from 0 to 4. Selection of schools and number of students from each school is given in appendix 1/additional file 1 (decision by the editorial board)

Food frequency data was obtained for the entire sample along with other nutritional status parameters that were objectives of a larger study. For the energy and macronutrient component of the study, a sub-sample of 500 students (~20% of the total sample), was selected by following a similar multi-stage sampling design as mentioned above. Four students out of these refused to participate in the interview questionnaire on the 24-hour dietary recall, thus giving a response rate of 99% for this aspect of the study.

### Ethical Considerations

Permission to conduct the study was obtained from the Ethical committees of the Ministry of Health and Ministry of Education. Inclusion of a student in the study was determined by parental consent. All permission letters were sent to parents 3-4 weeks prior to the study allowing them adequate time to take a decision. Confidentiality of information was assured to the parents and the children. Parents were informed if a student was found to have a nutritional health problem as determined by the different assessment methods that were part of the larger study.

### Data Collection

The study instrument was a questionnaire which included information on the age and gender of the child, a 24-hour dietary recall and food frequency. Age was recorded in completed years and verified by the researcher (NG) from school records. While the food-frequency questionnaire was self-administered by all the selected students in the classroom, information on the 24-hour dietary recall was obtained for a sub-sample by interviews that were conducted in a special room in the school premises. Interviews were conducted by a trained nurse, a nutritionist or one of the investigators (NG). Care was taken to conduct the interviews in a non-judgmental manner. For children ≤ 10 years of age, the interview for the 24-hour dietary recall was conducted in the presence of their mothers who were invited to attend the school on the day of the study. Most of the mothers in the girls' section attended the session but fewer mothers came in the boys' section. Mothers, who failed to attend the session, were contacted on telephone or home-visited by one of the interviewers to get data on the 24-hour dietary recall of their children. Effort was made to ensure that the child was present with his/her mother at home during the interview session. Data for the 24-hour recall was obtained for all food eaten on the previous day from wake-up to bed time. Information collected represented food consumed on a week-day. Week-end food intake may be variable and hence may not be representative of the usual dietary pattern for most days. Mothers and children were not informed about the method of data collection (24-hour dietary recall) prior to the study to avoid any bias which may have occurred due to change in the diet of children on the day before the interview.

During the interview, samples of local household dishes and utensils (different sizes of bowls, plates, cups, glasses, and spoons) were displayed to the child/mother. They were then shown pictures of common foods eaten in these dishes/utensils to indicate portion sizes consumed. One of the investigators (NG) had weighed different portions of various food items, placed them in the dishes/utensils and photographed them prior to the survey. After a student/mother as child proxy had indicated the portion size of food consumed, the corresponding weight of that food was recorded in the 24-hour dietary questionnaire. The latter had a list of 156 different food/drink items including: milk and dairy products, meat group, fast food types (local & international), bread and cereal group, mixed dishes eaten locally, vegetables and fruits, desserts and snacks, beverages (coffee & tea, juice, and fruit drinks), and other miscellaneous items. Some of these items were further classified, e.g.: type of milk (whole, low fat, or skim), type of meat eaten (boiled, fried, or grilled). There were open-ended questions for certain food items, e.g. type of fruit, vegetable and snack.

Mean energy and macronutrient intakes of the children were compared with the Dietary Reference Values (DRV) of the United Kingdom by age groups and gender [22]. Energy intake was compared to the Estimated Average Requirements (EARs) and macronutrient intake to the Reference Nutrient Intake (RNI) which is a value two notional standard deviations above EAR.

In addition, Energy percent (E%) from protein, carbohydrate and fat from total calories consumed, were assessed and compared to the DRVs recommended by the Committee on Medical aspects of Food Policy (COMA) [22].

To describe the potential effects of diet on serum lipid levels, dietary cholesterol levels and the Polyunsaturated to Saturated Fat Ratio (P: S ratio) were estimated. Dietary cholesterol intake of ≥ 300 mg and a P: S ratio of less than 1 was considered unacceptable [23].

### Food Frequency Data

The food items were grouped into 7 categories based on similarity of nutrient profiles [24]. These categories were: (1) meats & alternatives, including fish, chicken, red meat, eggs, and legumes; (2) milk & dairy products; (3) cereals, including rice, bread, cornflakes, and pasta; (4) fruits & vegetables including leafy and other vegetables; (5) sweets & snacks, including, cakes, chocolates, biscuits, candies, and crisps; (6) sauces & spreads, including ketchup, hot sauce, jams, and butter; (7) soft drinks, including all fizzy drinks (regular & diet), fruit juices, tea and coffee. Response categories were never, 1-2 times a week, 3-4 times a week, and 5-7 times a week (almost daily).

### Data Entry and Statistical Analysis

Dietary analyses were performed using Nutritionist 5 (First Data Bank, version 1.6, 1998) [25]. It is an American food data base and nutrient analysis software that includes over 17,000 food items with approximately 80 nutrients and nutrient factors. It was also possible through this software to enter the ingredients of Bahraini food recipes and obtain their nutrient content. Averages of energy and nutrient data by age groups and gender were then exported into Microsoft Excel 2000 (version 9.0). Statistical analyses were performed using Statistical Package for the Social Sciences (version 11.5, SPSS, 2002, SPSS Inc., Chicago, IL) for frequencies, means, standard deviations, and other descriptive and inferential statistics [26]. The chi-square, Kruskal-Wallis and Mann-Whitney tests were used where appropriate [27] A P-value less than 0.05 was considered to indicate statistical significance and a value of less than 0.01 as strongly significant.

### Validity

Height and weight of these children was measured and BMI calculated as an objective of the larger study. Details of methods for the anthropometric measurements are described elsewhere [28]. We adopted the criteria of the World Health Organization for overweight status [29]. A comparison between the energy intake and the BMI status of students showed that the mean energy intake of the non-overweight (1775 Kcal/day) was significantly lower than those at-risk of overweight or overweight/obese (2182 Kcal/day) (p < 0.05). With these findings one can make some general assumptions on the validity of the students' reported energy intake.

Quality controls to improve reliability and validity included 1) a protocol that specified exact techniques for interviewing, recording and calculating results; 2) standardizing food portion sizes in commonly used dishes/utensils for quantification of foods and beverages consumed; 3) use of the American food data base, Nutritionist 5 [25]; 4) rigorous training of interviewers prior to the study and 5) involvement of mothers in the interview for children ≤10 years old.

## Results

The age of the students ranged from 6 to 18 years. Mean ages of the girls and boys were 12.63 (SD 3.27) and 12.45 (SD 3.39) respectively.

### 1. Energy and Macronutrient Intake

Overall, the mean energy intake of Bahraini students ranged between 82.5% and 103% of the recommended EARs for different age groups and gender. The percentage of mean energy intake when compared to the EAR values decreased with increasing age of boys and girls. Overall the mean energy intake in boys was significantly higher than that in girls (p < 0.01) and this pattern was consistent at all ages. (Table 1).

**Table 1:** Daily Intake of Energy and Macronutrients of Bahraini students (Boys = 256 & Girls = 240) compared to the Dietary Reference Values (EAR, RNI) for the United Kingdom according to age groups ^22^

	Boys							Girls						
	
Macronutrient	7 - 10 Years (n = 84)		11-14 Years (n = 97)		15-18 Years (n = 75)		All ages	7 - 10 Years (n = 70)		11-14 Years (n = 91)		15-18 Years (n = 79)		All ages
	
	Mean (SD)	Standard (%)^1^	Mean (SD)	Standard (%)	Mean (SD)	Standard (%)	Mean (SD)	Mean (SD)	Standard (%)	Mean (SD)	Standard (%)	Mean (SD)	Standard (%)	Mean (SD)
Energy (kcal)	1851.1**^d ^**(347.9)	1,970 (94.0)	2101.3**^d ^**(548.1)	2,220 (94.7)	2367**^d ^**(548.7)	2,755 (85.9)	2097.1**^b ^**(530.3)	1793.4 (472.7)	1,740 (103.1)	1822.3 (447.7)	1,845 (98.8)	1739.9 (386.3)	2,110 (82.5)	1786.8**^b ^**(435.8)

Protein (gm)	70.7 **^d^**(18.2)	28.3 (249.8)	83.4**^d ^**(24.2)	42.1 (198.1)	91.4**^d ^**(23.2)	55.2 (165.6)	81.6**^b ^**(23.5)	68.8 (19.2)	28.3 (243.1)	68 (19.9)	41.2 (165.0)	67 (20.6)	45.0 (148.9)	67.9**^b ^**(19.9)

%Kcals Proteins	15.3 (2.5)		15.9 (2.4)		15.5 (2.4)		15.6 (2.5)	15.4 (2.2)		15 (2.8)		15.4 (3)		15.2 (2.7)

Carbohydrate (gm)	241.5**^d ^**(49.4)		274**^d ^**(69.4)		319.9**^d ^**(79)		276.8**^b ^**(73.3)	229.2 (62)		236.7 (65.3)		230.1 (54.1)		232.4**^b ^**(60.7)

%Kcals Carbohydrates	52.3**^c ^**(5.2)		52.5**^c ^**(5.1)		54 **^c^**(5.2)		52.9 (5.2)	51.2 (5)		52 (6)		53 (5.6)		52.1 (5.6)

Fat, total (gm)	68.8**^d ^**(16.7)		76.6**^d ^**(25)		82.2**^d ^**(22.8)		75.7**^b ^**(22.5)	68.7 (21.8)		68.8 (19.6)		63.3 (16.9)		67**^b ^**(19.5)

%Kcals Fats	33.3**^d ^**(3.8)		32.5**^d ^**(4)		31.1**^d ^**(3.9)		32.4 (4)	34.3**^c ^**(4.1)		33.9**^c ^**(4.3)		32.7**^c ^**(4)		33.6 (4.2)

Cholesterol (mg)	260.3 (100.6)		300.8 (121.6)		299.8 (167)		287.2**^b ^**(131.6)	265 (121.2)		243.5 (119.6)		229.3 (123.7)		245.1**^b ^**(121.7)

Saturated Fat (gm)	22.1 (7.3)		23.1 (8.4)		24.4 (8.6)		23.2**^a ^**(8.1)	23.1**^c ^**(9.3)		22.1**^c ^**(7.3)		19.5**^c ^**(6.2)		21.6**^a ^**(7.7)

%kcals Saturated	10.6**^d ^**(2.3)		9.9**^d ^**(2.1)		9.3**^d ^**(2.4)		9.9 (2.3)	11.4**^d ^**(2.2)		10.9**^d ^**(2.3)		10.1**^d ^**(2.2)		10.8 (2.3)

Monounsaturated Fat (gm)	19.9**^d ^**(5.9)		21.2**^d ^**(7.5)		24**^d ^**(7)		21.6**^b ^**(7)	19 (7.2)		18.9 (6.7)		17.7 (5.7)		18.5**^b ^**(6.5)

%kcals Monosaturated	9.6 (1.9)		9 (1.9)		9.1 (1.5)		9.3 (1.8)	9.4 (1.8)		9.2 (2)		9.1 (1.9)		9.3 (1.9)

Polyunsaturated Fat (gm)	12.2 (3.5)		14 (5.6)		13.5 (4.3)		13.3**^b ^**(4.7)	12.4 (4.5)		12.7 (5.4)		11.8 (4.6)		12.3**^b ^**(4.9)

%kcals Ployunsaturated	5.9**^d ^**(1.4)		6**^d ^**(1.7)		5.2**^d ^**(1.3)		5.7 (1.5)	6.3 (1.9)		6.2 (1.9)		6.1 (1.9)		6.2 (1.9)

MFA 18:1, Oleic (gm)	7.8 (4.1)		7.8 (3.8)		8.6 (4.7)		8.0**^a ^**(4.2)	7.6 (4.9)		7.1 (3.7)		6.8 (3.5)		7.1**^a ^**(4)

PFA 18:2, Linoleic (gm)	3.9**^c ^**(2.1)		4.9**^c ^**(3.1)		4.6**^c^**(2.1)		4.5 (2.6)	4.3 (2.7)		4.3 (2.4)		3.9 (2)		4.2 (2.4)

PFA 18:3, Linolenic (gm)	0.3 (0.1)		0.3 (0.2)		0.3 (0.1)		0.3 (0.1)	0.3 (0.2)		0.3 (0.1)		0.3 (0.1)		0.3 (0.2)

PFA 20:5, EPA (gm)	0 (0)		0 (0)		0 (0)		0 (0)	0 (0)		0 (0)		0 (0.1)		0 (0)

PFA 22:6, DHA (gm)	0 (0.1)		0.1 (0.1)		0 (0)		0 (0.1)	0 (0.1)		0 (0.1)		0.1 (0.1)		0.1 (0.1)

Dietary Fiber, total (gm)	12.4**^c ^**(5.6)		13.6**^c ^**(5.6)		14.8**^c ^**(6.1)		13.5**^a ^**(5.8)	12 (5.4)		12.2 (5.8)		13 (6.5)		12.4**^a ^**(5.9)

Sugar, total (gm)	93.2**^d ^**(31.1)		98**^d ^**(34.5)		114.6**^d ^**(43.5)		101.3**^b ^**(37.3)	87.1 (30.7)		93.8 (40.1)		85.5 (35.6)		89.1**^b ^**(36.1)

Sucrose (gm)	22.7 (11.3)		22.3 (12.3)		25.2 (15)		23.3 (12.8)	17.5 (9.2)		16.5 (9.7)		18.3 (11.8)		17.4 (10.3)

Lactose (gm)	8.3 (4.5)		6.5 (4.3)		6 (5.9)		6.9**^b ^**(4.9)	9 (5.6)		6.2 (4)		5.4 (4.7)		6.7**^b ^**(4.9)

P:S **^2^**	0.6 (0.2)		0.6 (0.2)		0.6 (0.2)		0.6 (0.2)	0.6 (0.2)		0.6 (0.2)		0.6 (0.2)		0.6 (0.2)

1: Standard Reference Values (UK): EAR for energy and RNI for Protein, (percentage of standard) [22]; 2: Polyunsaturated fat to Saturated fat ratio

**a **p < 0.05; **b **p < 0.01 (Mann-Whitney test) Significantly different between genders

**c **p < 0.05, **d **p < 0.01 Significantly different across age groups

Table 1 shows that the mean intake of protein exceeded the RNI at all ages and in both genders. Children 7-10 years old consumed about 2.5 times more protein relative to RNI values whereas adolescent boys and girls consumed 1.5 times the RNI values. The Protein E% from was 15.6% in boys and 15.2% in girls and these findings were close to the recommendations set by COMA [22] on energy intake from Protein (15%). The Protein E% did not change significantly with increasing age in boys and girls.

The mean daily intake of carbohydrate was significantly higher in boys (276.8 g ± 73.3) than in girls (232.4 g ± 60.7) (p < 0.01) (Table 1). This trend remained consistent for all age groups. In boys, carbohydrate intake increased significantly with age up to 18 years (p < 0.01) but in girls, intake increased up to 14 years and decreased thereafter. The carbohydrate E% was 52.9% in boys and 52.1% in girls which is close to the recommendations of COMA [22] for energy intake from carbohydrate (50%). While in girls the E% from carbohydrate did not change with age, in boys, it significantly increased with age (p < 0.05).

The mean daily intake of total sugars (intrinsic and extrinsic sugars) of 101.3 g ± 37.3 for boys and 89.1 g ± 36.1 for girls was high according to the maximum recommended intake of 60 g/day by the DRV of UK [22]. Sugar consumption increased while lactose intake decreased with age. The mean daily intake of dietary fiber was significantly higher in boys (13.5 g ± 5.8) than in girls (12.4 g ± 5.9) (p < 0.05) (Table 1). For adolescents these values apparently fell short of the current recommendations for fiber intake [30-32].

The mean daily total fat intake for boys (75.7 g ± 22.5) was higher than that for girls (67 g ± 19.5) (p < 0.01) and this trend remained consistent in all age groups. Mean fat intake decreased in late adolescent girls. Overall, boys had significantly higher mean daily intakes of monounsaturated and polyunsaturated fats than girls (p < 0.01; p < 0.01) (Table 1). The mean P: S ratio was 0.6 ± 0.2 for both boys and girls. The mean daily intake of cholesterol was 287.2 mg ± 131.6 for boys and 245.1 mg ± 121.7 for girls (p < 0.01). The Total fat E% was 32.4% for boys and 33.6% for girls and this was slightly lower than the recommendations set by COMA [22] for energy intake from fat (35%). The Total fat E% did not change with age in girls but significantly decreased with age in boys (p < 0.01) (Table 1). The E% from saturated fat were slightly higher in girls (10.8%) than in boys (9.9%) (p < 0.01). The E% from saturated and polyunsaturated fats significantly decreased with age in boys (p < 0.01; p < 0.05)

According to the recommendations of the DRV standards of COMA for children above 5 years, saturated fat and total fat should not provide more than 11% and 35% of total dietary energy respectively [22]. We found that 50.2% and 37.8% of girls and boys respectively, exceeded these limits for saturated fat and 46.9% and 39.2%, respectively for total fats. Diets of 35.8% girls and 50.2% boys contained more than 300 mg of cholesterol per day which is the suggested upper limit [33,34].

### 2. Mean Energy, E% of Macronutrients, Sugar, Fiber and P:S Ratio by Diets having Different Levels of Total Fat Contribution to Energy

Analysis was done to determine the association of mean energy intake, E% of macronutrients, dietary cholesterol, sugar and dietary fiber with diets having Total fat E% at 3 levels (< 30%, between 30-37%, and ≥ 38%). Table 2 shows that while Carbohydrate E%, sugar and dietary fiber were significantly higher in the less than more dense-fat diets (p < 0.01; p < 0.01; p < 0.01), a reverse trend was observed for fatty acids (saturated, monounsaturated, and polyunsaturated) in the diet of girls and boys. Though not statistically significant, intake of dietary cholesterol (mg/1000 Kcal) was also found to increase with increasing density of total fat in diets. The P: S ratio remained below 1 at all 3 levels of fat-dense diets.

**Table 2:** Distribution of Mean Energy, Macronutrient Densities, P: S Ratio, Mean sugar and Dietary fiber by levels of Total Fat dense diets in Bahraini Girls (n = 240) and Boys (n = 256)

	Total Fat Dense Diets (%kcal)				
		
Nutrient variables	< 30%	30-37%	≥ 38%	Total	^1^ P-value
		
	Mean	Mean	Mean	Mean	
		
	n = 42	n = 166	n = 32	n = 240	
**Girls**					

**Energy (Kcal)**	1645.605	1794.366	1932.672	1786.774	0.032 **^a^**

**%Kcals from Proteins**.	15.068	15.15	15.788	15.221	0.589

**%Kcals from Carbohydrates**	58.502	51.978	44.348	52.102	0.000 **^b^**

**%Kcals from Fats**	27.594	33.815	40.428	33.608	0.000**^b^**

**%kcals from Sat**	8.764	10.975	12.583	10.802	0.000**^b^**

**%kcals from Monosaturated fat**	7.464	9.301	11.374	9.256	0.000**^b^**

**%kcals from Polyunsaturated fat**	4.878	6.183	7.996	6.196	0.000**^b^**

**Cholesterol (mg)**	195.155	245.783	307.002	245.085	0.001**^b^**

**Cholesterol, mg/1000 kcal**	116.94	136.705	160.123	136.369	0.010 **^a^**

**Sugar, Total (gm)**	99.969	89.42	73.222	89.106	0.003**^b^**

**Sucrose (gm)**	19.578	17.079	16.265	17.408	0.502

**Dietary Fiber, total (gm)**	14.558	12.246	10.284	12.389	0.009**^b^**

**P:S Ratio^3^**	0.586	0.587	0.659	0.596	0.121

**Boys**					

**Energy (Kcal)**	2008.984	2127.582	2146.195	2097.07	0.426

**%Kcals from Proteins**.	15.417	15.632	15.87	15.592	0.722

**%Kcals from Carbohydrates**	58.007	51.714	44.609	52.855	0.000**^b^**

**%Kcals from Fats**	27.537	33.432	40.236	32.375	0.000**^b^**

**%kcals from Sat**	8.638	10.08	13.358	9.947	0.000**^b^**

**%kcals from Monosaturated fat**	7.933	9.528	11.661	9.265	0.000**^b^**

**%kcals from Polyunsaturated fat**	4.939	5.993	6.301	5.733	0.000**^b^**

**Cholesterol (mg)**	271.479	287.863	336.387	287.238	0.156

**Cholesterol, mg/1000 kcal**	136.601	135.939	159.025	137.921	0.194

**Sugar, Total (gm)**	107.408	101.689	76.853	101.29	0.004**^b^**

**Sucrose (gm)**	24.52	23.028	21.096	23.279	0.541

**Dietary Fiber, total (gm)**	15.229	12.762	14.258	13.544	0.005**^b^**

**P:S Ratio^2^**	0.615	0.622	0.498	0.611	0.039 **^a^**

**1 **Significantly different across categories of total fat dense diets categories **a **p < 0.05; **b **p < 0.01 (Kruskal-Wallis test)

**2 **Polyunsaturated-Saturated Ratio

### 3. Food Frequency

**Figure 1:**
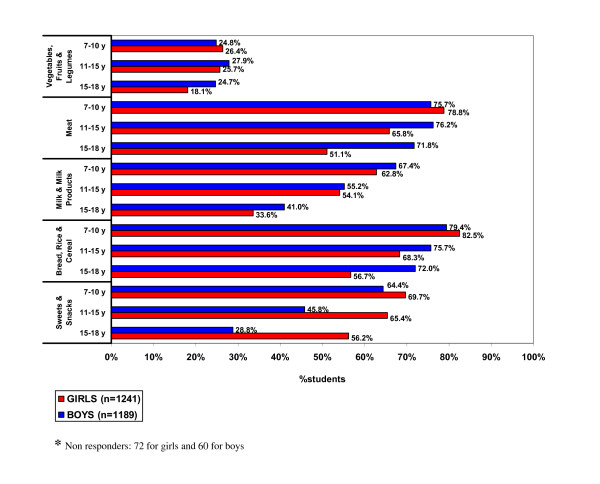
**Daily consumption of food groups by Bahraini students of different age groups**.

Table 3 shows that bread and cereal, meat group and sweets and snacks were the most commonly consumed daily food items among students. Only half of the children were consuming milk and its products and one-fourth of them were taking vegetables and fruits on a daily basis. However, regular soda drinks were consumed daily by approx. 50% of the children. Daily consumption of sweets and snacks was significantly more common in girls (64.2%) than in boys (47.5%) (p < 0.01). On the other hand, meat, milk and its products and tea/coffee were more popular with boys than girls (p < 0.01; p < 0.05; p < 0.01). Figure 1 shows that daily consumption of most food items in girls and milk and its products in boys decreased with age but intake of sweets and snacks increased with age in both genders.

**Table 3:** Frequency of consumption of Food Items among Bahraini Girls (n = 1241) and Boys (n = 1190)*

Food	Almost daily 5-7 times/w		3-4 times/w		1-2 times/w		Never	
	
	**No**.	%	**No**.	%	**No**.	%	**No**.	%
**GIRLS**								

Milk & Milk Products	635	51.2	366	29.5	231	18.6	9	0.7

Bread, Rice & Cereal	870	70.1	256	20.6	114	9.2	1	0.1

Fruits	443	35.7	464	37.4	308	24.8	26	2.1

Fruit Juices	328	26.4	371	29.9	455	36.7	87	7

Vegetables Leafy	404	32.6	370	29.8	366	29.5	101	8.1

Vegetables Others	146	11.8	432	34.8	543	43.8	120	9.7

Meat (Fish, Poultry, Meat)	822	66.2	303	24.4	110	8.9	6	0.5

Eggs	111	8.9	396	31.9	627	50.5	107	8.6

Legumes: lentils, beans...	35	2.8**^c^**	115	9.3	733	59.1	358	28.8

Sweets & Snacks: cakes, chips, candies,...	797	64.2**^d^**	300	24.2	134	10.8	10	0.8

Miscellaneous: tea & coffee	289	23.3	318	25.6	411	33.1	223	18

Miscellaneous: sauce & spreads	214	17.2	356	28.7	497	40	174	14

Soda Drinks: regular	600	48.3	322	25.9	254	20.5	65	5.2

Soda Drinks: diet	19	1.5**^d^**	29	2.3	116	9.3	1077	86.8

**BOYS**								

Milk & Milk Products	659	55.4**^a^**	356	29.9	170	14.3	5	0.4

Bread, Rice & Cereal	904	76**^b^**	227	19.1	56	4.7	3	0.3

Fruits	492	41.3**^a^**	425	35.7	258	21.7	15	1.3

Fruit Juices	359	30.2	358	30.1	399	33.5	74	6.2

Vegetables Leafy	400	33.6	340	28.6	340	28.6	110	9.2

Vegetables Others	176	14.8	397	33.4	520	43.7	97	8.2

Meat (Fish, Poultry, Meat)	889	74.7**^b^**	234	19.7	65	5.5	2	0.2

Eggs	107	9**^a^**	396	33.3	621	52.2	66	5.5

Legumes: lentils, beans,...	30	2.5	139	11.7	730	61.3	291	24.5

Sweets & Snacks: cakes, chips, candies,...	565	47.5	353	29.7	262	22	10	0.8

Miscellaneous: tea & coffee	412	34.6**^b^**	267	22.4	331	27.8	180	15.1

Miscellaneous: sauce & spreads	178	15	396	33.3	443	37.2	173	14.5

Soda Drinks: regular	629	52.9**^b^**	322	27.1	197	16.6	42	3.5

Soda Drinks: diet	17	1.4	25	2.1	67	5.6	1081	90.8

Difference between genders: **a **p < 0.05; **b**: p < 0.01 (within gender); **c **p < 0.05, **d **p < 0.01 (between genders)

* Non responders: 72 for girls and 59 for boys

## Discussion

The current research is the first study to evaluate the energy and macronutrient intake in Bahraini school children. It was part of a larger study on nutritional status of children and hence it was possible to validate some of the findings. Though there is a possibility of under or over-reporting of food consumption in a 24-hour recall study for various reasons related to knowledge, memory and interview situation [35], reported data on energy intake when compared to Body Mass Index of the students showed a significant trend in the expected direction. This finding allowed us to make some assumption on the validity of the responses.

There may have been some misreporting of data for boys ≤10 years old whose mothers did not participate in the study. The study data has its drawbacks of a one-time 24-hour dietary recall that may not be representative of the usual diet for an individual child. However, it does represent estimations of the average dietary intake of a group because the means of data collection are robust and unaffected by within-person variation [35].

The mean energy intakes of Bahraini children and adolescents were higher than the EAR standards as well as values reported from the US, some European countries such as, Switzerland, Spain, Germany and the UK [36-40] and some Asian countries such as China, Greece and Bangladesh [37,41,42]. Mean energy intake of Bahraini children is comparable to data of adolescents from France [36]. High intake of energy by the Bahraini students is a cause of serious concern. Could excessive calorie consumption have contributed to the high prevalence of overweight status found in Bahraini children of this study [43]?

The average protein intake was well in excess of the RNI at all ages. Although there are methodological differences between the present study and dietary studies from the UK [39], Sweden [44] and the USA (Bogalusa Heart Study) [45], Bahraini children were observed to consume more protein than children from these western countries.

Protein is required for growth of children. However, if it is consumed in excess of needs, it is diverted to the energy pathway, or if it is above caloric needs it is metabolized into fat [46]. In fact, a high protein intake early in life could increase the risk of adiposity later in life [47].

The mean daily intake of sugars (intrinsic and extrinsic) was high as per DRV recommendations [22] suggesting an increased risk of dental caries, obesity and its related health problems in Bahraini children. While sugar consumption increased with age due to increasing intake of chocolates, candies and sugared soft drinks, the intake of lactose which helps in calcium absorption, decreased with age due to reduced intake of milk (Table 2). It is a cause of concern that around 50% of the study population consumed at least 1 regular soda drink per day. Research has shown that daily consumption of a 12-oz sugared soda drink has been associated with a 0.18-point increase in a child's BMI and a 60% increased risk of obesity. Over-consumption is a problem when energy is ingested in liquid form; moreover, these drinks represent energy added to, without displacing other dietary intake [48].

Besides weight gain, increased sugar consumption in Bahraini children is likely to decrease their HDL cholesterol, increase LDL cholesterol, triglycerides, blood glucose and insulin concentrations, factors which are related to Coronary Heart Disease mortality [49]. Further, research suggests that high sugar intake may lead to nutritional inadequacy of micronutrients especially those of Vitamin A, C, B12, Folate, Calcium, Phosphorus, Magnesium and Iron [50].

Dietary fiber is a part of carbohydrate and is necessary for normal laxation; it may also help prevent future risk of cardiovascular disease, some cancers, and adult-onset diabetes [31,32]. A reasonable goal for dietary fiber intake during childhood and adolescence may be approximately equivalent to the age of the child +5 grams per day and a safe range is age +5 to +10 g/day [30-32].

Fiber intake by the Bahraini adolescents apparently fell short of the current recommendations and may be considered inadequate for optimal health promotion and chronic disease prevention [31]. This is apparently due to low consumption of fresh fruits and vegetables as observed in the food frequency data of the children and possibly due to insufficient quantities of other sources of fiber such as whole grains, legumes, and high fiber cereals. Indeed, findings of this survey were consistent with those of a recent national nutrition survey conducted on Bahraini adults, 50% of whom were not consuming fruits and vegetables on a daily basis [9].

Regional studies have also shown that daily consumption of fruits and vegetables were uncommon among Omani adolescent girls and young females from the United Arab Emirates [15,51].

Nutrition messages in Bahraini school children should emphasize that foods high in starches (polysaccharides; e.g., bread, pasta, cereal, potatoes) are recommended over sugar (monosaccharide's and disaccharides) as per dietary guidelines of the American Heart Association [49]. Moreover, foods that are sources of complex carbohydrates (whole grains) as well as nutrient-fortified and enriched starches, such as cereals should be the major sources of calories in the diet.

A higher mean total fat intake by boys than girls might be explained by their more frequent consumption of fat-rich foods such as meats, eggs, and whole milk and dairy products. The mean total fat intake was lower among Bahraini adolescent girls compared to their younger counterparts. Two possible reasons could explain these finding. Older girls are more likely to be preoccupied with body image [52], and hence are less keen to accurately report foods higher in fat and sugar, which they perceive to be "unhealthy"; alternatively some of them were under a controlled diet at the time of the survey to reduce their body weight and hence were cautious in fat consumption. These possibilities need further exploration since they were not investigated in the present study.

The mean E% of macronutrients among Bahraini boys and girls were close to the current dietary recommendations set by COMA [22] and comparable with values of E% in the diet of French adolescents (48.1% carbohydrate, 36.9% fat, 15% protein) [36] and the UK children and adolescents (51.5% carbohydrate, 35.4% fat, 13.1% protein for boys; and 51% carbohydrate, 35.9% fat, 13.1% protein, for girls) [39]. However, a higher total fat E% was found in American children and adolescents (38%) [53] and Greek adolescents (40.25%) [37] compared to their Bahraini counterparts of this study.

The E% from fat and fatty acids for young people is generally compared with the DRVs for adults since the significance of any long-term effects are less well established for children than for adults and hence reference values have not been formulated separately for children. However it is suggested that the dietary patterns of fat intake recommended for adults should be appropriate for children from the age of five years [22].

It was encouraging to note that the average E% of total fat and saturated fat among Bahraini students were equal to or lower than the recommended Dietary Values [22]. Whether this suggests a lower risk of ischemic heart disease in the long term has yet to be established. Bahraini mean values of E% for saturated fat were also lower than the US mean values of 12% (NHANES III) for children aged 2 to 19 years [38] and UK mean values of 14% for children aged 7 to 18 years [39].

The mean E% of monounsaturated fat among Bahraini students was lower than the DRV value of 13% and that of the US children (12.5%) and UK children (11.8%) [22,38,39]. Bahraini E% values of polyunsaturated fat were close to the DRV of 6.5%, as well as to the mean values of boys and girls in the UK (6%) [22,39]. However, the P:S ratio of 0.6 for both girls and boys, was lower than the usually recommended value of 1, suggesting higher consumption of saturated fat compared to polyunsaturated sources [23]. This situation is not encouraging considering saturated fatty acids in the diet raise plasma total and LDL cholesterol while polyunsaturated fatty acids (particularly linoleate in corn, safflower, sunflower, and soybean oils) and monounsaturated fatty acids (principally oleate in olive oils), lower these blood lipids [33].

The mean daily intake of dietary cholesterol among Bahraini boys was higher as per DRV standards of ≤ 300 mg/day [22] and is a cause of concern. A higher dietary cholesterol intake in boys than girls is possibly due to their higher consumption of cholesterol-rich foods such as meats, eggs, milk and other dairy products.

While the mean E% of total fat and saturated fat as well as mean cholesterol intake in the diet of Bahraini children was at an acceptable level as per DRV recommendations, a substantial proportion of the students exceeded prudent dietary recommendations. There are data to suggest, that children with high levels of serum cholesterol may have an increased risk of having high serum cholesterol as adults [54]. Dietary fat intake in children is of interest because of concerns about the atherosclerotic process which begins in childhood and increases cardiovascular disease risk factors including hypertension [53,54]. Current dietary findings suggest that one-third to half of the Bahraini children may be at increased risk of cardiovascular disease and mortality in their adult life.

Though the mean E% of total and saturated fat in the study population was acceptable, increasing values of saturated fat and cholesterol and decreasing values of fiber intake in high fat dense diets

(> 30%) indicates the higher potential of an atherogenic effect and cardiovascular disease risk in those consuming such diets. P: S values of all 3 fat-dense diets were lower than the usually recommended value of 1 [23], suggesting that diets with apparently 'unfavorable' lipid profiles are not a phenomenon exclusive to the high fat group. These data suggest that, in addition to the quantitative aspects, the qualitative nature of dietary fat intake of school-aged children and adolescents deserves attention.

Sugar intake was significantly more in those on low than high fat dense diets. It is not unreasonable to speculate that students on low fat dense diets might compensate by increasing the use of sucrose to maintain energy requirements. In the Bogalusa Heart Study, children whose E% from total fat was less than 30% consumed more carbohydrates (mainly sucrose) than children who ate high-fat food [55]. To avoid increased calorie consumption and hence weight gain, high sugar and nutrient-poor foods should not be a substitution for reduced fat intake as per AHA guidelines [49].

The food frequency data showed that a small selection of foods made up the total diet for most children. While milk is a good source of protein and calcium necessary during the growth phase of children, there is concern about its infrequent consumption especially during adolescence. Infrequent intake of fruits and vegetables reflects lack of protective and healthy foods in the diet of Bahraini children. Moreover, high intake of regular soda drinks, sweets and snacks will contribute to surplus empty calories with an increased risk for obesity and its consequences.

## Conclusions

Low intake of fruits and vegetables and dietary fiber, high sugar intake and high Energy % of saturated fat and dietary cholesterol by a substantial proportion of Bahraini children is likely to increase their risk of obesity and cardiovascular diseases in later life. Therefore, nutrition education programs in schools should emphasize the importance of healthy balanced diets and the risks of consuming empty calories. Parents too should be provided information on nutrients essential for the growth and health maintenance of children as well as dietary prevention of diseases. Moreover, food industries have a potential to change the eating habits of our children. They should provide healthier food choices and introduce attractive ways to market these new choices to appeal to the children. It is well known that the foundation for good health in adulthood is laid during childhood and adolescent years.

## Competing interests

The authors declare that they have no competing interests.

## Authors' contributions

NG designed the study, drafted the manuscript and supervised the data collection and statistical analysis. PR conceived the study, participated in its design and coordination, helped to interpret the data and draft the final form of the manuscript. Both authors read and approved the final manuscript.

## Supplementary Material

Additional file 1 **Number of Schools Selected from each Region (no. of students)**. This file contains data on number of schools selected from each Region and number of students selected from each school. Click here for file
