# Supplementary material for: Energy and macronutrient intake and dietary pattern among school children in Bahrain: a cross-sectional study
Source: Nutr J. 2011 Jun 5;10:62. doi: 10.1186/1475-2891-10-62 (PMC3123629; doi:10.1186/1475-2891-10-62)
Supplement: Additional file 1 — Number of Schools Selected from each Region (no. of students). This file contains data on number of schools selected from each Region and number of students selected from each school. [file 1475-2891-10-62-S1.DOC]

**Appendix 1:** Number of Schools Selected from each Region (no. of students)

| **School** | **Total** | **Region** | | | | | | | | | | | |
| --- | --- | --- | --- | --- | --- | --- | --- | --- | --- | --- | --- | --- | --- |
|  |  | **Hamad Town** | **Western** | **Riffa** | **Isa Town** | **Central** | **Sitra** | **Northern** | **Jidhafs** | **Manama** | **Muharraq** | | **Hidd** |
|  | **No. of School (no. of students^†^)** | | | | | | | | | | | | |
| **Girls** |  |  |  |  |  |  |  |  |  |  |  |  | |
| Primary | 29 (716) | 3 (97) | 2 (66) | 3 (40) | 3 (29) | 2 (45) | 2 (5) | 2 (54) | 3 (75) | 4 (149) | 4 (117) | 1 (39) | |
| Intermediate | 13 (278) | 1 (58) | 0**^‡^** | 1 (52) | 1 (9) | 2 (7) | 1 (18) | 2 (21) | 1 (27) | 1 (19) | 2 (61) | 1 (6) | |
| Secondary | 12 (319) | 1 (38) | 0**^‡^** | 2 (15) | 1 (98) | 0**^‡^** | 0**^‡^** | 1 (10) | 1 (24) | 2 (101) | 2 (33) | 0**^‡^** | |
| **Total** | **54 (1313)** | **5 (193)** | **2 ( 66)** | **6 (107)** | **5 (136)** | **4 (52)** | **3 (23)** | **5 (85)** | **5 (126)** | **7 (269)** | **8 (211)** | **3 (45)** | |
|  |  |  |  |  |  |  |  |  |  |  |  |  | |
| **Boys** |  |  |  |  |  |  |  |  |  |  |  |  | |
| Primary | 29 (655) | 3 (92) | 2 (61) | 3 (68) | 3 (92) | 2 (45) | 2 (64) | 2 (23) | 3 (15) | 4 (86) | 4 (98) | 1 (11) | |
| Intermediate | 13 (305) | 1 (35) | 1 (23) | 1 (33) | 1 (28) | 1 (20) | 1 (54) | 1 (5) | 1 (26) | 2 (32) | 2 (42) | 1 (7) | |
| Secondary | 12 (289) | 1 (37) | 1 (7) | 1 (13) | 1 (12) | 0**^‡^** | 1 (19) | 0**^‡^** | 2 (30) | 2 (128) | 1 (43) | 0**^‡^** | |
| **Total** | **54 (1249)** | **5 (164)** | **4 (91)** | **5 (114)** | **5 (132)** | **3 (65)** | **4 (137)** | **3 (28)** | **6 (71)** | **8 (246)** | **7 (183)** | **2 (18)** | |
| **All*** | **108 (2562)** | **10 (357)** | **6 (157)** | **11 (221)** | **10 (268)** | **7 (117)** | **7 (160)** | **8 (113)** | **11 (197)** | **15 (515)** | **15 (394)** | **5 (63)** | |

**†** Selection of students in proportion to the number of educational levels in each school

**‡** Some regions did not have secondary schools

***** The regions for 32 children was missing
